# Micro- and Macro-Vascular Disease in Systemic Sclerosis and Very Early SSc (VEDOSS): Results from a Monocentric Observational Study

**DOI:** 10.3390/biomedicines14030607

**Published:** 2026-03-09

**Authors:** Vincenzo Zaccone, Silvia Contegiacomo, Silvia Agarbati, Chiara Paolini, Carolina Clementi, Matteo Mozzicafreddo, Silvia Svegliati, Lorenzo Falsetti, Devis Benfaremo, Gianluca Moroncini

**Affiliations:** 1 PhD Program in Human Health, Department of Clinical and Molecular Sciences, Marche Polytechnic University, Via Tronto 10/A, 60126 Ancona, Italy; vincenzozaccone@gmail.com; 2 Internal Medicine Residency Program, Marche Polytechnic University, Via Tronto 10/A, 60126 Ancona, Italy; silviacontegiacomo@gmail.com; 3 Department of Clinical and Molecular Sciences, Marche Polytechnic University, Via Tronto 10/A, 60126 Ancona, Italy; s.agarbati@staff.univpm.it (S.A.); c.paolini@staff.univpm.it (C.P.); c.clementi@pm.univpm.it (C.C.); m.mozzicafreddo@staff.univpm.it (M.M.); s.svegliati@staff.univpm.it (S.S.); l.falsetti@staff.univpm.it (L.F.); g.moroncini@univpm.it (G.M.); 4 Clinica Medica, Department of Internal Medicine, Marche University Hospital, Via Conca 71, 60126 Ancona, Italy

**Keywords:** systemic sclerosis, VEDOSS, microvascular involvement, macrovascular disease, atherosclerosis, endothelial dysfunction, arterial disease

## Abstract

**Background**: Systemic sclerosis (SSc) is characterized by endothelial dysfunction leading to progressive vascular injury and fibrosis. While microvascular involvement is well established as an early disease feature, macrovascular disease has been historically underrecognized and poorly investigated in very early disease stages. Integrated assessments across the SSc spectrum, including very early diagnosis of systemic sclerosis (VEDOSS), remain limited. **Methods**: In this cross-sectional observational study, patients with established SSc, VEDOSS, and primary Raynaud’s phenomenon (PRP) were prospectively enrolled between October 2023 and April 2025. Participants underwent comprehensive microvascular and macrovascular evaluation, including nailfold videocapillaroscopy, multisegmental arterial Doppler ultrasound (carotid, aortic, and lower limb districts), flow-mediated dilation, and measurement of endothelial biomarkers (vascular cell adhesion molecule 1 (VCAM-1), intercellular adhesion molecule 1 (ICAM-1), and circulating endothelial cells (CECs)). Traditional cardiovascular risk was estimated using Systematic Coronary Risk Estimation 2 (SCORE2). **Results**: Sixty-two female subjects were included (34 SSc, 14 VEDOSS, and 14 PRP). Microvascular abnormalities followed the expected disease continuum, with capillaroscopic changes present in 57% of VEDOSS and 91% of SSc patients. Although SCORE2 estimates and carotid intima–media thickness were comparable across groups, macrovascular abnormalities were more frequent in SSc (52.9%) and VEDOSS (50%) compared with PRP (21.4%). VCAM-1, ICAM-1, and CEC levels were significantly increased in SSc compared with PRP, whereas no significant differences were observed between VEDOSS and PRP. **Conclusions**: These findings support a unified micro- and macro-vascular disease model in SSc and demonstrate that macrovascular involvement is detectable already in the VEDOSS phase. Conventional cardiovascular risk scores underestimate the true vascular burden, highlighting the need for disease-specific risk stratification tools integrating vascular imaging and endothelial biomarkers.

## 1. Introduction

Systemic sclerosis (SSc) is a chronic connective tissue disease characterized by immune system activation, microvascular dysfunction, and progressive fibrosis of the skin and internal organs [1]. Although fibrosis has long been considered the hallmark of SSc [2], extensive evidence now identifies vascular injury as an early and central pathogenic event that drives subsequent tissue remodeling and organ involvement [3]. Microvascular impairment arises at disease onset, persists throughout the clinical course, and contributes substantially to morbidity and mortality [4].

Vascular involvement affects both the microcirculation and macrocirculation, producing a broad spectrum of clinical manifestations. Microvascular dysfunction underlies Raynaud’s phenomenon, usually the earliest and most common feature, followed by digital ulcers, telangiectasias, and ischemic tissue injury. Vasculopathy of internal organs can lead to severe complications, including pulmonary arterial hypertension (PAH), scleroderma renal crisis, and gastric antral vascular ectasia. These manifestations emphasize the central role of vascular dysfunction in disease progression [3].

Macrovascular involvement, historically underappreciated, has gained increasing recognition in recent years. Ultrasonographic and hemodynamic studies have demonstrated increased carotid intima–media thickness, arterial stiffness, multisegmental atherosclerotic plaques, and altered peripheral hemodynamics, suggesting accelerated atherosclerosis in SSc [5]. A large meta-analysis including more than six million individuals documented significantly elevated risks of stroke, myocardial infarction, venous thromboembolism, and peripheral arterial disease, indicating a systemic macrovascular burden not fully explained by traditional cardiovascular risk factors [6]. The persistence of this excess risk after adjustment for conventional variables supports the contribution of disease-specific inflammatory and autoimmune mechanisms [7].

Mechanistic studies show that microvascular and macrovascular involvement share common pathogenic determinants. Histopathology across vascular territories reveals convergent features, including endothelial activation or injury, intimal proliferation, medial thickening, and perivascular fibrosis [8]. These findings support a unified vascular framework in which the micro- and macro-circulation are affected through shared immune-mediated mechanisms [9]. Early disease is dominated by functional abnormalities such as vasospasm, endothelial activation, and increased permeability, clinically reflected in Raynaud’s phenomenon, puffy fingers, and capillaroscopic changes characteristic of very early diagnosis of SSc (VEDOSS) [8]. VEDOSS represents a pre-classification phase of the disease characterized by Raynaud’s phenomenon associated with early clinical and/or serological features suggestive of evolving SSc. These may include puffy fingers, SSc-specific autoantibodies, or microvascular abnormalities at nailfold capillaroscopy. Identification of VEDOSS enables recognition of patients at risk of progression before fulfillment of ACR/EULAR classification criteria and provides a unique window for studying early pathogenic mechanisms, including vascular alterations [8]. With disease progression, structural remodeling may lead to obliterative microangiopathy, ischemic complications, and advanced organ involvement.

Despite advances in understanding SSc vasculopathy, significant gaps of knowledge persist. Most clinical studies have evaluated micro- or macro-vascular involvement separately, and integrated analyses across the full disease spectrum—from VEDOSS to established SSc—remain scarce [8]. Furthermore, the interplay between micro- and macro-angiopathy and their role in disease progression are insufficiently defined.

In this study, we explored whether structural abnormalities of medium- and large-sized arteries—specifically consistent with the development of atherosclerotic disease in typical vascular territories—may represent part of a broader vascular involvement in SSc. We also aimed to evaluate the potential role of immune-mediated endothelial dysfunction in the development and progression of these atherosclerotic alterations. In this context, the term “macrovascular involvement” refers to imaging-detected structural abnormalities compatible with atherosclerotic disease.

Furthermore, we aimed to provide a comprehensive assessment of micro- and macro-vascular involvement in VEDOSS and established SSc by integrating endothelial function testing, multisegmental arterial ultrasonography, and circulating endothelial biomarkers.

## 2. Materials and Methods

### 2.1. Study Design and Population

A cross-sectional observational study was conducted to evaluate integrated microvascular and macrovascular involvement in patients with SSc. Between October 2023 and April 2025, consecutive adult female participants (>18 years) attending the Scleroderma Unit of Clinica Medica, Marche University Hospital, were prospectively enrolled. Participants could be included if they met the following criteria: (a) patients with diffuse (dcSSc) or limited (lcSSc) cutaneous SSc fulfilling the 2013 ACR/EULAR classification criteria [10], and (b) patients with VEDOSS according to published criteria [8], not yet meeting ACR/EULAR classification. VEDOSS classification was based on published criteria allowing classification in the presence of Raynaud’s phenomenon together with SSc-specific autoantibodies and/or puffy fingers, even in the absence of a definite scleroderma capillaroscopic pattern. Individuals with PRP followed at our institution constituted the control group. Exclusion criteria included life expectancy < 6 months (defined as severe non-SSc comorbid illness likely to limit short-term survival), active acute infection, known malignancy, known PAH, previous scleroderma renal crisis, current pregnancy, or inability to provide informed consent. PAH and prior SRC were excluded to avoid inclusion of patients with advanced organ-specific vascular complications that could confound the evaluation of early systemic vascular changes.

The study was approved by the Comitato Etico Regionale delle Marche (CERM; protocol number 2020107-OR) and performed in accordance with the Declaration of Helsinki. All participants received detailed information about the study procedures and provided written informed consent prior to enrollment.

### 2.2. Clinical Assessment

At enrollment, all participants underwent a standardized clinical evaluation, including:

- General clinical and cardiovascular risk assessment: age, hypertension, dyslipidemia, diabetes mellitus, smoking status. The 10-year cardiovascular risk was estimated using SCORE2 for individuals < 70 years and SCORE2OP for those ≥70 years, with region-specific charts for Italy, in accordance with ESC 2021 guidelines [11].
- Concomitant therapies: including cardiovascular (CV), vasoactive, and immunomodulatory drugs.
- Disease-specific variables: disease duration from the first non-Raynaud’s symptom, active or previous digital ulcers, previous amputations, presence of interstitial lung disease (ILD), pulmonary function tests (PFTs), modified Rodnan Skin Score (mRSS), EUSTAR Activity Index (EUSTAR-AI), and SCTC Damage Index (SCTC-DI).

### 2.3. Instrumental Assessment

All instrumental assessments were performed during the same study visit to ensure methodological consistency and minimize intra-individual variability.

Nailfold videocapillaroscopy was performed to classify microangiopathic patterns based on EULAR–CAP definitions [12] by a single experienced operator (S.C.). Flow-mediated dilation (FMD) of the brachial artery was assessed according to current evidence-based recommendations by a single experienced operator (V.Z.) [13]. Patients fasted ≥8 h, avoided caffeine/nicotine for ≥12 h, and discontinued short-acting nitrates ≥24 h or calcium channel blockers when clinically feasible. Examinations were performed in the supine position after 15 min of rest. The right brachial artery was imaged longitudinally (2–15 cm above the antecubital fossa) using a 11–17 MHz linear probe. After baseline diameter measurement, ischemia was induced by inflating a forearm cuff to 250 mmHg for 5 min, followed by rapid deflation. Post-stimulus diameter was measured at 60 s. FMD% was calculated as: FMD% = [(D_post − D_base)/D_base] × 100.

A comprehensive arterial ultrasound evaluation, including the carotid district, abdominal aorta, and lower limbs, was performed by a single experienced operator (V.Z.).

Carotid district. Carotid intima–media thickness (IMT) was measured at the common carotid artery, the carotid bifurcation, and the internal carotid artery, using a high-resolution linear probe (L9–3 MHz). IMT was classified as normal (<0.9 mm), intima–media thickening (0.9–1.4 mm), non-stenotic atherosclerosis (>1.4 mm), hemodynamically significant stenosis, or occlusion.

Abdominal aorta. Longitudinal and transverse scans from the diaphragm to the iliac bifurcation were performed using a 3.5–5 MHz convex probe. Findings were classified as normal, wall irregularities, non-stenotic atherosclerosis, hemodynamically significant stenosis, or occlusion. Aortic caliber was categorized as normal (≤2.5 cm), ectasia (2.5–3.0 cm), aneurysm (>3.0 cm), or thrombosed aneurysm.

Lower limbs. Femoro-popliteal and tibial arteries were assessed in the supine position using a L9–3 MHz probe and classified as normal, diffuse calcifications, non-stenotic atherosclerosis, hemodynamic stenosis, or occlusion.

The ankle-brachial index (ABI) was calculated, with values 0.9–1.3 considered normal, <0.9 indicative of peripheral arterial disease, and >1.3 suggestive of severe medial arterial calcification.

Standard transthoracic echocardiography, including estimation of systolic pulmonary arterial pressure (sPAP), derived by adding right ventricular end-diastolic pressure to tricuspid regurgitation velocity (TRV), was performed by a single experienced operator (L.F.).

### 2.4. Sample Collection

Blood samples were obtained at the time of enrollment. Peripheral blood samples were collected in EDTA tubes and processed within 4 h from bleeding. Twelve milliliters of blood was collected through a venous drawing in EDTA tubes. The first 3 mL of blood from the venipuncture was not used for cell analysis because of the contaminating presence of endothelial cells derived from the vessel wall. In order to assess serum biomarkers, peripheral venous blood was collected in tubes containing clot activator or trisodium citrate. Serum, specifically platelet-poor plasma (PPP), was obtained within 60 min of sampling by centrifugation at 1200 g for 10 min at room temperature and then aliquoted and frozen at −80 °C (Marche Biobank, Ancona, Italy) until analysis. Sera with more than two freeze–thaw cycles were excluded.

### 2.5. Flow Cytometry and ELISA Analysis

Briefly, a volume of peripheral blood containing 20 × 10^6^ leukocytes underwent an erythrocyte-lysis step, with 1X Pharm Lyse solution (Becton-Dickinson, Franklin Lakes, NJ, USA) according to the manufacturer’s instructions. Cells were stained with different monoclonal antibodies (mAbs) for the detection of CECs: anti-CD31*FITC (Becton-Dickinson), anti-CD105*BB700 (Becton-Dickinson), anti-CD34 *PE-cyanine 7 (Becton-Dickinson), anti-CD133* allophycocyanin (Becton-Dickinson), anti-CD45* allophycocyanin-cyanine 7 (Becton-Dickinson), and FV510* V500 (Becton-Dickinson) [14]. To ensure the specific identification of target cells and rule out platelet contamination, an anti-CD61 (Becton-Dickinson) was included in the panel to gate out CD61-positive events.

Single staining and fluorescence minus one (FMO) control were performed for all panels to set proper compensation and define positive signals. In order to identify rare cells like human peripheral CECs, it was mandatory to acquire a huge number of cells [15], that is, of the order of several million per sample.

**Figure 1:**
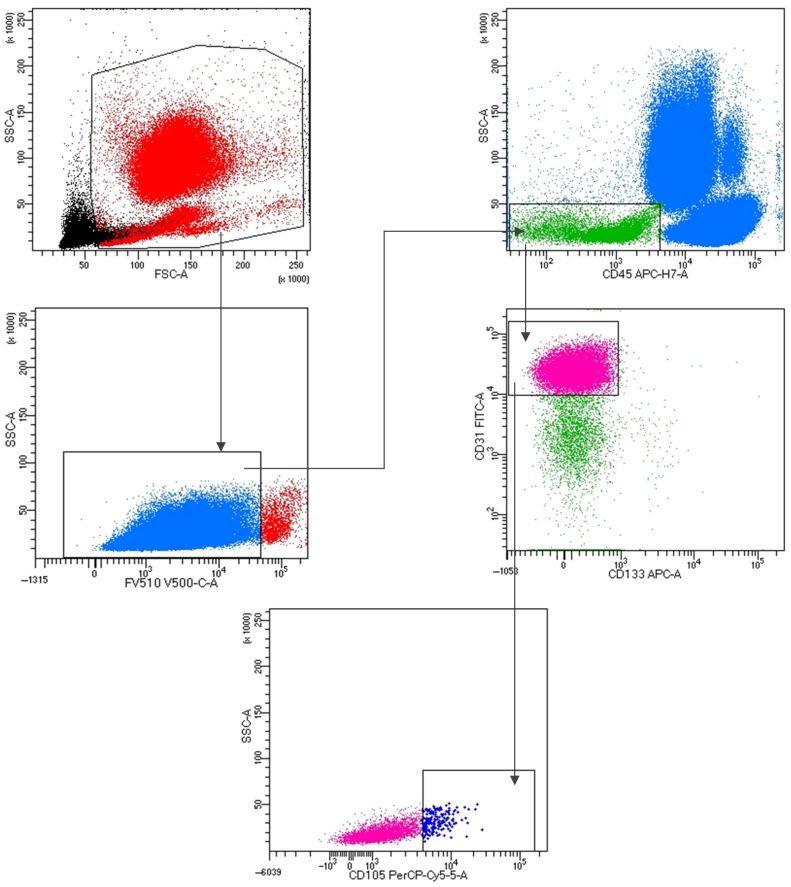
Gating strategy for the identification of CECs. Debris and dead cells were excluded by the use of an electronic gate, containing cells identified by a viability marker, i.e., FV510. CECs were identified on the basis of the expression of CD45, CD31, CD133, and CD105: CECs were defined as CD45dim, CD31+, CD133−, and CD105+. FSC: forward scatter; SSC: side scatter.

For phenotype analysis, 3.1 × 10^6^ events/sample with lympho-monocyte morphology (SSC/FSC dot plot) were acquired by 8-color 3-laser BD FACSLyric (Becton-Dickinson) and analyzed using BD FACSDiva v 9 (Becton-Dickinson). A specific gating strategy was employed to enumerate viable CECs, defined by the following immunophenotype: FV510-, CD45dim, CD31+, CD133-, and CD105+. The gating strategy is shown in Figure 1.

An unstained control was used to subtract background autofluorescence. CEC counts were always expressed as a percentage of peripheral blood total events. Instrument performance and data reproducibility were checked according to the manufacturer’s instructions, before each evaluation.

Serum levels of ICAM-1 and VCAM-1 were analyzed using a commercially available ELISA kit following the manufacturer’s instructions (Human sICAM-1 Elisa Kit, Human VCAM Elisa Kit, Thermo Fisher, Eugene, OR, USA).

### 2.6. Statistical Analysis

Continuous variables were reported as mean and standard deviation (SD) or as median and interquartile range (IQR), according to their distribution. Differences between groups were evaluated using the *t*-test for two-group comparisons with normally distributed variables, or one-way ANOVA for comparisons involving more than two groups. For non-normally distributed variables, the Mann–Whitney U test (two groups) or the Kruskal–Wallis test (more than two groups) was applied. Categorical variables were expressed as absolute frequencies and percentages. Differences between groups were assessed using the chi-square test. A two-sided *p*-value ≤ 0.05 was considered statistically significant.

## 3. Results

### 3.1. Population Characteristics

A total of 62 subjects were enrolled during the study period and stratified into 3 groups: 34 patients with SSc, including 21 with limited cutaneous SSc (lcSSc) and 13 with diffuse cutaneous SSc (dcSSc), 14 patients classified as VEDOSS, and 14 individuals with PRP, who served as the control group. All participants were female. The median disease duration in the SSc group was 6 (IQR 3.25–11) years. Most patients with SSc were either anti-topoisomerase (47%) or anti-centromere (29.4%) positive and had early (38.2%) or active (41.1%) scleroderma pattern at capillaroscopy. Fifteen out of thirty-four patients had a history of digital ulcers. Median age was comparable across groups (SSc: 56 (IQR 49.6–61.8); VEDOSS: 48 (20 IQR 36–56); PRP: 48.5 (IQR 41–56.8); *p* = 0.08). Table 1 summarizes the main clinical and demographic characteristics of the study population.

### 3.2. Clinical and Instrumental Assessment of Macrovascular Disease

Clinical and instrumental findings related to macrovascular disease are summarized in Table 1 and Table 2. The three groups did not differ regarding the prevalence of CV risk factors. Accordingly, the 10-year global cardiovascular risk, estimated using the SCORE2 and SCORE2OP, was comparable among groups (SSc 2.15%, VEDOSS 1.5%, and PRP 1.6%; *p* = 0.68), indicating that neither patients with established SSc nor those with VEDOSS exhibited increased estimated cardiovascular risk relative to PRP controls.

While the proportion of subjects with carotid intima–media thickening (IMT > 0.9 mm), considered an imaging surrogate of overall cardiovascular risk, was comparable across groups (SSc 11.7%, VEDOSS 7.1%, and PRP 7.1%; *p* = 0.829), multisegmental arterial ultrasound revealed a higher prevalence of structural abnormalities compatible with atherosclerosis in SSc patients and, to a similar extent, in those with VEDOSS, compared with the PRP control group. Specifically, the presence of plaques, stenosis, or occlusions in the carotid, aortic, or lower limb arterial districts, as well as aneurysmal dilations > 3 cm, was more frequent in SSc (52.9%) and VEDOSS (50%) compared with PRP controls (21.4%), although the difference did not reach statistical significance (*p* = 0.12). Likewise, the ABI was more frequently abnormal in SSc patients (21.4%) than in those with VEDOSS (7.14%) or PRP (0%), though the difference was not statistically significant (*p* = 0.183).

The assessment of endothelial dysfunction by FMD showed a higher prevalence of impaired values (FMD < 7%) in the PRP group (71%) compared with the SSc (44.1%) and VEDOSS (28.6%) groups, although this difference did not reach statistical significance (*p* = 0.068). No differences in sPAP were observed among the three groups (*p* = 0.737), although the TRV was higher in SSc than PRP controls (2.44 (IQR 2.35–2.63) vs. 2.31 (2.13–2.43), *p* = 0.04).

### 3.3. Biomarkers Analysis

**Figure 2:**
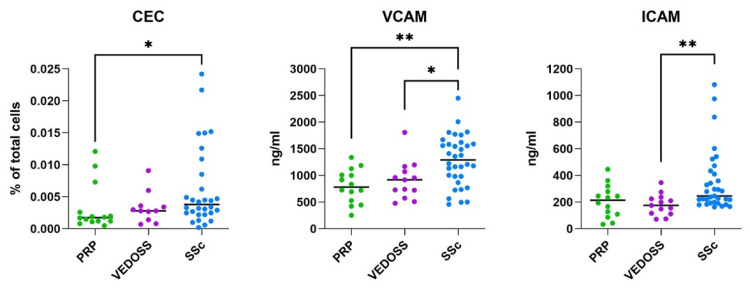
Markers of endothelial activation and injury in SSc, VEDOSS, and control subjects with PRP. Percentages of CECs (first panel) and ICAM-1 and VCAM-1 levels (second and third panels) assessed, respectively, by flow cytometry and the ELISA test. *p*-values, calculated by the Kruskal–Wallis test, are indicated in the figure, * *p* < 0.05 and ** *p* < 0.001.

Serological analyses demonstrated significantly increased levels of endothelial activation markers in SSc patients compared with control subjects (Figure 2 and Table 2). Notably, VCAM-1 levels were significantly higher in SSc (1291 (IQR 984–1589) ng/mL) than in PRP (780 (IQR 569–1007) ng/mL), while both ICAM-1 and VCAM-1 were elevated in SSc (246 (IQR 199–411) ng/mL and 1291 (IQR 984–1589) ng/mL, respectively) compared with VEDOSS (176 (IQR 116–225) ng/mL and 918 (IQR 725–1072) ng/mL, respectively).

Similarly, CEC counts, considered a direct indicator of endothelial injury [16], were significantly increased in SSc patients compared with PRP controls (*p* < 0.05), whereas differences between SSc and VEDOSS did not reach statistical significance (Figure 2).

### 3.4. Atherosclerosis and Biomarker Analysis

In the overall assessment of patients with SSc and VEDOSS, subjects with atherosclerosis detected by multisegment Doppler ultrasound were generally older (56 vs. 50 years, *p* = 0.044) and had higher circulating levels of VCAM-1 (1223.14 vs. 982.51 ng/mL, *p* = 0.033), suggesting a potential role for this biomarker in identifying individuals at increased risk of structural large-vessel disease. No significant differences were observed in circulating levels of ICAM-1 or CECs between the two groups. These subjects showed comparable FMD, ABI, and IMT values compared with patients without atherosclerosis. Interestingly, global cardiovascular risk, as estimated using SCORE2 risk charts, was also comparable between the two groups in this analysis. No significant differences were observed in terms of disease characteristics.

## 4. Discussion

This observational study investigated the extent of microvascular and macrovascular involvement in patients with established SSc and individuals with VEDOSS, compared with a control group with PRP.

Central to both microvascular and macrovascular pathology is the endothelium, which acts as a pathogenic hub integrating inflammatory, mechanical, and metabolic stimuli [17]. Autoimmune, oxidative, or infectious triggers induce a persistently activated endothelial phenotype characterized by reduced nitric oxide bioavailability, increased endothelin-1 production, and upregulation of adhesion molecules, such as VCAM-1 and ICAM-1. This dysfunctional state promotes vasoconstriction, inflammation, thrombosis, and vascular leakage [18]. In parallel, impaired vascular repair, reflected by defective angiogenesis, reduced endothelial progenitor cell function, and endothelial-to-mesenchymal transition [19], drives maladaptive vascular remodeling and fibrosis, positioning SSc as a model of systemic endothelial dysfunction [20].

Within this pathogenic framework, microvascular disease has long been recognized as a defining and early feature of SSc. In contrast, the involvement of the macrocirculation has historically been more controversial, with earlier views considering SSc a predominantly “pure” microangiopathy that largely spares large- and medium-sized arteries. However, over the past two decades, increasing evidence has challenged this notion [6]. Doppler ultrasound, angiography, and functional studies have demonstrated a higher prevalence of peripheral arterial disease, carotid plaques [5], ulnar and radial artery occlusions, and impaired flow-mediated dilation in SSc compared with controls [21]. These macrovascular abnormalities have also been associated with digital ischemia and ulcer recurrence [22]. Nevertheless, most existing studies have focused exclusively on patients with established SSc, often with long disease duration and accumulated vascular damage [6]. Data on early or preclinical stages are sparse, and macrovascular involvement has generally been interpreted as a late consequence of chronic inflammation, fibrosis, or accelerated atherosclerosis.

Our findings challenge this paradigm by demonstrating a higher atherosclerotic burden already affecting individuals in the VEDOSS phase, at levels comparable to those observed in established SSc. The detection of macrovascular abnormalities in approximately half of VEDOSS patients—clearly exceeding those observed in PRP—supports the concept that macrovascular damage is an early event in the disease course rather than a secondary, late-stage complication. To our knowledge, the systematic assessment of macrovascular involvement specifically in VEDOSS represents a novel contribution to the literature and fills a significant gap in early SSc research.

A key unresolved issue is whether the detected plaques represent (i) accelerated atherosclerosis, (ii) disease-specific vascular remodeling affecting larger vessels, or (iii) an overlap phenotype. Morphologically, plaques are compatible with classical atherosclerosis. However, their presence in VEDOSS—despite comparable SCORE2 risk—raises the possibility that immune-mediated endothelial injury may contribute to structural arterial changes beyond conventional atherosclerotic mechanisms. This reinforces the inadequacy of traditional cardiovascular risk algorithms in capturing the excess vascular burden associated with SSc and VEDOSS, particularly when early macrovascular damage appears to arise independently of conventional risk factors and precedes overt endothelial activation. These observations support the need for disease-specific cardiovascular risk models that integrate immunologic and vascular determinants beyond traditional atherosclerotic risk factors.

Analysis of endothelial dysfunction markers revealed differential endothelial activation across the disease spectrum. VCAM-1 and ICAM-1 levels were significantly higher in SSc than in VEDOSS and PRP and CECs were also increased in SSc compared with PRP. These findings indicate a higher burden of endothelial activation in established SSc. Importantly, the increased macrovascular and atherosclerotic burden observed in VEDOSS was not accompanied by a parallel increase in endothelial activation markers or CECs. This suggests that early macrovascular alterations may develop independently of measurable endothelial dysfunction, at least as captured by the biomarkers assessed in this study. The apparent dissociation between early macrovascular involvement and microvascular or endothelial injury in VEDOSS highlights a complex and possibly staged vascular pathogenesis, in which different vascular compartments may be affected asynchronously. This mismatch underscores the need to further elucidate endothelial biology in the earliest phases of disease and to identify complementary mechanisms—such as immune-mediated vascular remodeling, altered shear stress responses, or subclinical inflammation—contributing to early macrovascular injury.

Overall, the findings support the “unified vascular framework”, in which SSc is conceptualized as a systemic vascular disease characterized by a continuous micro- and macro-vascular injury process driven by persistent endothelial dysfunction [9]. While persistent endothelial dysfunction appears to dominate later disease stages, our data suggest that early macrovascular damage may precede overt endothelial activation, particularly in the VEDOSS phase. The dissociation between conventional cardiovascular risk estimation and the true vascular burden observed in SSc and VEDOSS highlights the need to develop personalized risk stratification tools incorporating vascular imaging and biomarkers of endothelial activation and injury. Markers such as VCAM-1, ICAM-1, and CECs may serve as promising candidates for identifying patients at risk for vascular progression, particularly in established disease.

This study has several limitations, including the small sample size, single center design, and absence of longitudinal follow-up, all of which may limit statistical power and generalizability. Although median age did not differ significantly across groups, exploratory analyses suggested that structural abnormalities were more frequent in older individuals. Given the limited sample size, formal age-adjusted modeling was not feasible and represents an important limitation. The lack of adjustment for vasoactive therapies, disease activity, and comorbidities represents an additional limitation. Finally, the biomarker panel employed captures only part of the complex pathogenetic landscape underlying SSc vasculopathy.

In conclusion, this study provides new insights into the evolutionary trajectories of vascular injury in SSc [9]. These findings support the hypothesis that structural large-vessel abnormalities may emerge early in the SSc disease continuum, although their precise relationship with classical atherosclerosis versus disease-specific vasculopathy remains to be clarified. At the same time, the lack of concomitant endothelial activation in VEDOSS points to a dissociation between microvascular/endothelial dysfunction and macrovascular injury in the earliest stages of disease. Future research should include larger, multicenter cohorts and longitudinal designs to clarify the temporal relationship between endothelial dysfunction and macrovascular damage, and to determine whether early macrovascular abnormalities predict progression to definite SSc or major cardiovascular and thromboembolic events.

## Figures and Tables

**Table 1:** Demographic and clinical characteristics of the study cohort.

Characteristic	All (n = 62)	SSc (n = 34)	VEDOSS (n = 14)	PRP (n = 14)	*p*
**Gender (female)**	62 (100%)	34 (100%)	14 (100%)	14 (100%)	1.00
**Age (years)**	54 (44, 61)	56 (49.6–61.8)	48 (36–56)	48.5 (41–56.8)	0.08
**Disease subset (dcSSc/lcSSc)**	N/A	13 (21%)/21 (34%)	N/A	N/A	N/A
**Disease duration (years)**	5.0 (2.0, 10.0)	6 (3.25–11)	2 (1–6.25)	N/A	N/A
**Autoantibodies (%)**					
Negative	15 (24%)	0	1	14	N/A
Anti-centromere	17 (27%)	10	7	0	N/A
Anti-topoisomerase I	19 (31%)	16	3	0	N/A
ANA or other autoantibodies	11 (18%)	8	3	0	N/A
**mRSS**	N/A	4 (1, 7)	N/A	N/A	N/A
**Lung involvement (%)**	N/A	22 (65%)	N/A	N/A	N/A
**FVC (%)**	N/A	105 (89, 117)	N/A	N/A	N/A
**DLCO (%)**	N/A	66 (54, 76)	N/A	N/A	N/A
**Capillaroscopic findings**					
Normal or non-specific abnormalities	22 (35%)	3	6	14	N/A
Early scleroderma pattern	22 (35%)	13	8	0	N/A
Active scleroderma pattern	14 (23%)	14	0	0	N/A
Late scleroderma pattern	4 (6.5%)	4	0	0	N/A
**Active digital ulcers**	N/A	6 (17%)	N/A	N/A	N/A
**Previous digital ulcers**	N/A	15 (42%)	N/A	N/A	N/A
**Digital ulcers ever**	N/A	15 (42%)	N/A	N/A	N/A
**Lower limbs ulcers**	N/A	2 (3.2%)	N/A	N/A	N/A
**Previous limb amputation**	N/A	1 (1.6%)	N/A	N/A	N/A
**EUSTAR AI**	N/A	1.5 (1–2.13)	N/A	N/A	N/A
**SCTC DI**	N/A	2 (2–4)	N/A	N/A	N/A
**sPAP (mmHg)**	29 (27, 32)	28.5 (27–32)	28.5 (26.5–31.8)	29 (24.5–30.8)	0.737
**TRV (m/s)**	2.40 (2.26, 2.60)	2.44 (2.35–2.63)	2.38 (2.05–2.43)	2.31 (2.13–2.43)	0.04
**CV risk factors (%)**					
Active smoker	4 (6.6%)	2	1	1	0.858
Former smoker	8 (13%)	4	3	1	0.858
Diabetes	6 (9.7%)	5	0	1	0.274
Arterial hypertension	14 (23%)	11	2	1	0.116
Dyslipidemia	15 (24%)	8	1	6	0.087
**SCORE2 or SCORE2OP (%)**	2.10 (0.90, 3.50)	2.15 (1–3.58)	1.51 (0.8–3.37)	1.6 (0.92–3.17)	0.680
**Treatments (%)**					
CCB	15 (24%)	11	2	2	0.255
Bosentan	15 (24%)	11	4 *	0	0.054
MMF	11 (18%)	11	N/A	N/A	N/A
Nintedanib	5 (8.1%)	5	N/A	N/A	N/A
Statin	11 (18%)	7	1	3	0.497
ACE/ARB	12 (19%)	10	0	2	0.055
Oral anticoagulants	1 (1.6%)	1	0	0	0.658
Antiplatelet	2 (3.2%)	2	0	0	0.427
Biologics	4 (6.5%)	4	N/A	N/A	N/A
Prostacyclin Analogs	14 (23%)	12	1	1	N/A

Abbreviations: ACE, angiotensin-converting enzyme; AI, activity index; ANA, antinuclear antibodies; ARB, angiotensin II receptor blockers; CCB, calcium channel blockers; CV, cardiovascular; dcSSc, diffuse cutaneous SSc; DI, damage index; DLCO, diffusing capacity of the lung for carbon monoxide; EUSTAR, European Scleroderma Trials and Research group; FVC, forced vital capacity; lcSSc, limited cutaneous SSc; MMF, mycophenolate mofetil; mRSS, modified Rodnan Skin Score; sPAP, systolic pulmonary artery pressure; SCORE2, Systematic Coronary Risk Evaluation 2; SCORE2OP, Systematic Coronary Risk Evaluation 2—Older Persons; SCTC, Scleroderma Clinical Trials Consortium; TRV, tricuspid regurgitation velocity. * In VEDOSS patients, bosentan was prescribed for severe Raynaud’s phenomenon with ischemic risk.

**Table 2:** Main instrumental and laboratory parameters comparing SSc, VEDOSS, and control subjects with PRP.

Characteristic	SSc (n = 34)	VEDOSS (n = 14)	PRP (n = 14)	*p*
**FMD, median (IQR)**	7.85 (2.77–12.7)	8.35 (5.79–11.1)	2.72 (0.00–9.22)	0.188
**Reduced FMD (%)**	15	4	10	0.068
**IMT, median (IQR)**	0.6 (0.6–0.77)	0.6 (0.51–0.6)	0.6 (0.6–0.74)	0.140
**High IMT (%)**	4	1	1	0.829
**ABI, median (IQR)**	1.08 (0.94–1.22)	1.13 (1.02–1.20)	1.11 (1–1.25)	0.731
**Reduced ABI (%)**	6	0	1	0.183
**Atherosclerotic plaques (%)**	18	7	3	0.126
**CEC (%)**	0.00380 (0.002–0.007)	0.00280 (0.002–0.003)	0.00175 (0.001–0.002)	0.046
**ICAM (ng/mL), median (IQR)**	246 (199–411)	176 (116–225)	215 (114–270)	0.005
**VCAM (ng/mL), median (IQR)**	1291 (984–1589)	918 (725–1072)	780 (569–1007)	0.001

Abbreviations: ABI, ankle–brachial index; CECs, circulating endothelial cells; FMD, flow-mediated dilation; ICAM, intercellular adhesion molecule; IMT, intima–media thickness; VCAM, vascular cell adhesion molecule.

## Data Availability

The data that support the findings of this study are available from the corresponding author upon reasonable request.

## Abbreviations

The following abbreviations are used in this manuscript:

ABI	Ankle-Brachial Index
ACE	Angiotensin-Converting Enzyme
AI	Activity Index
ANA	Antinuclear Antibodies
ARB	Angiotensin II Receptor Blockers
CCB	Calcium Channel Blockers
CECs	Circulating Endothelial Cells
CV	Cardiovascular
dcSSc	Diffuse Cutaneous SSc
DI	Damage Index
DLCO	Diffusing Capacity of the Lung for Carbon Monoxide
EUSTAR	European Scleroderma Trials and Research group
EUSTAR-AI	EUSTAR Activity Index
FMO	Fluorescence Minus One
FMD	Flow-Mediated Dilation
FVC	Forced Vital Capacity
ICAM-1	Intercellular Adhesion Molecule 1
ILD	Interstitial Lung Disease
IMT	Intima–Media Thickening
IQR	Interquartile Range
lcSSc	Limited Cutaneous SSc
mAbs	Monoclonal Antibodies
MMF	Mycophenolate Mofetil
mRSS	Modified Rodnan Skin Score
PAH	Pulmonary Arterial Hypertension
PFTs	Pulmonary Function Tests
PPP	Platelet-Poor Plasma
PRP	Primary Raynaud’s Phenomenon
SCORE2	Systematic Coronary Risk Estimation 2
SCORE2OP	Systematic Coronary Risk Evaluation 2—Older Persons
SCTC	Scleroderma Clinical Trials Consortium;
SCTC-DI	SCTC Damage Index
SD	Standard Deviation
sPAP	Systolic Pulmonary Arterial Pressure
SSc	Systemic Sclerosis
TRV	Tricuspid Regurgitation Velocity
VCAM-1	Vascular Cell Adhesion Molecule 1
VEDOSS	Very Early Diagnosis of Systemic Sclerosis
